# Identification and Management of Substance Misuse in Patients Referred for Psychodynamic Psychotherapy: A Service Evaluation Project

**DOI:** 10.1192/bjo.2022.400

**Published:** 2022-06-20

**Authors:** Joshua Lusby, Kenny Chu, Shivanthi Sathanandan, Thomas Hillen

**Affiliations:** Camden and Islington NHS Foundation Trust, London, United Kingdom

## Abstract

**Aims:**

To evaluate and improve upon collection of data pertaining to substance misuse in opt-in questionnaires sent to patients accepted by a psychodynamic psychotherapy service in an inner London Trust, and to explore options for improving care for the population of patients identified as potentially having a substance misuse issue who are accepted by the service.

**Methods:**

An initial audit of all opt-in questionnaires was conducted between September 2019 and September 2020. In this analysis of 144 responses, 72 were found to have indicated that they had experience of a substance misuse disorder. Of these responses, 55% of answers made it possible to discern the substance of misuse, and 31% of answers allowed for differentiation between historic and current misuse. In response to these findings, the AUDIT-C was incorporated into the opt in questionnaire with data subsequently collected and analysed between December 2020 and February 2021. Subsequently, patients identified as having and AUDIT-C score indicating possible harmful or dependent drinking were offered interventions stratified by score with a clinician supervised by the substance misuse service

**Results:**

In the re-audit period of opt-in questionnaires, there were 31 respondents, of whom 90% indicated that they or a contact had been concerned about their substance use. Inclusion of the AUDIT-C allowed for differentiation between alcohol and other substances in 100% of cases, and in 71% of cases it was possible to discern current vs historic substance misuse. In the period of December 2020- December 2021, four patients accepted the offer of a brief intervention.

**Conclusion:**

It was felt that obtaining higher quality data pertaining to substance misuse in patients accepted for psychotherapy was beneficial in terms of being better able to understand needs of patients and also to guide clinical management. Furthermore, introduction of a pathway to guide interventions in cases identified as being potentially at risk appeared to be effective in addressing substance misuse within this population. It is hoped that this preliminary evaluation can guide further service model development within this psychotherapy department and within other services in order to better address the needs and improve access to services of patients with comorbid substance misuse disorders.

